# Supplementary material for: Functional Interaction of SKA and NDC80 Complexes at Kinetochores Promoting Anaphase Onset in Mitosis
Source: bioRxiv. 2026 May 24:2026.05.22.727258. Preprint. [Version 1] doi: 10.64898/2026.05.22.727258 (PMC13228555; doi:10.64898/2026.05.22.727258)
Supplement: 1 [file NIHPP2026.05.22.727258v1-supplement-1.pdf]

bioRxiv preprint doi: https://doi.org/10.64898/2026.05.22.727258; this version posted May 24, 2026. The copyright holder for this preprint (which was not certified by peer review) is the author/funder, who has granted bioRxiv a license to display the preprint in perpetuity. It is made available under a CC-BY 4.0 International license.

A.

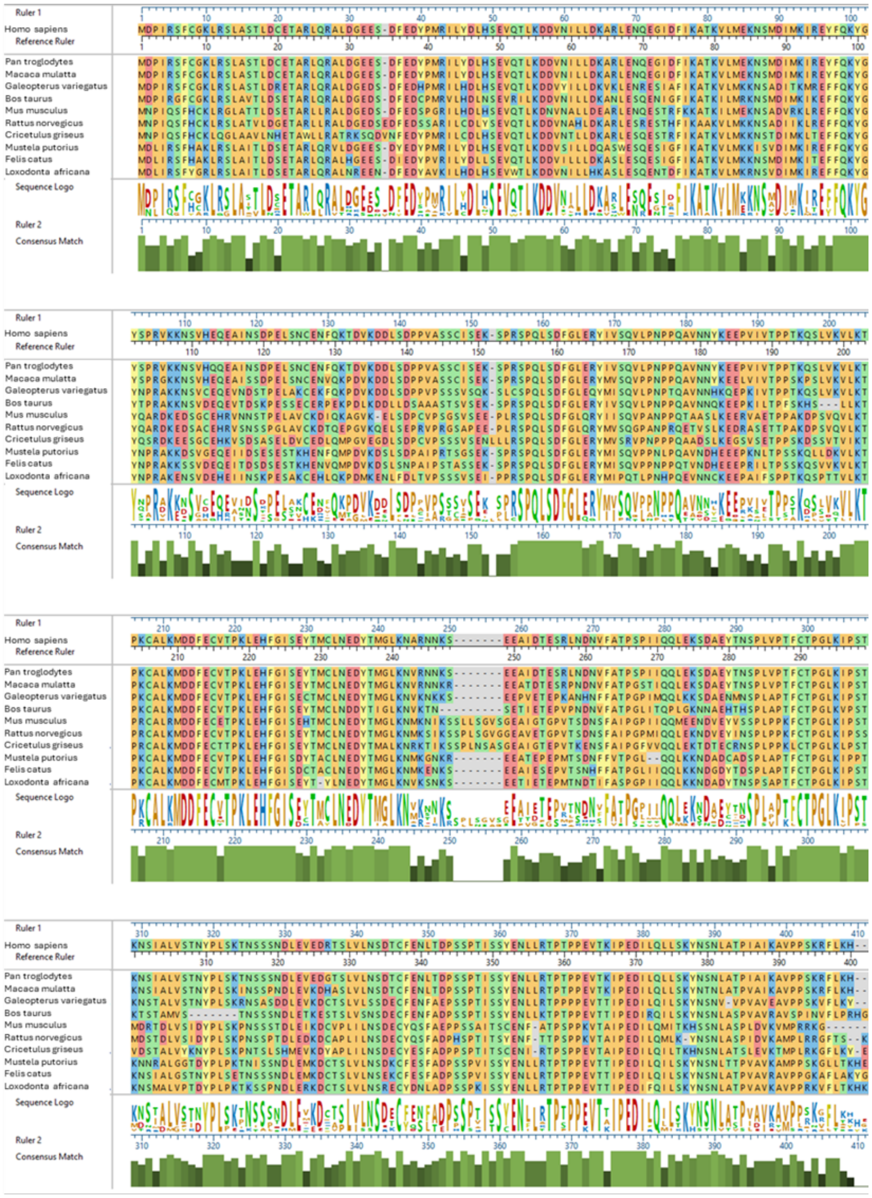

B.

Percent Identity/Percent Similar

		A	B	C	D	E	F	G	H	I	J	K
A	Homo sapiens		99.2	94.6	79.8	75.1	74.9	75.1	76.2	63.2	63.5	63.2
B	Pan troglodytes	99.5		94.6	79.8	74.9	74.6	75.1	76.2	63.0	63.2	63.0
C	Macaca mulatta	96.1	96.4		79.0	75.4	75.1	74.4	75.9	64.0	62.7	64.5
D	Galeopterus variegatus	88.6	88.9	87.0		75.1	75.1	74.4	74.9	64.5	64.2	62.7
E	Felis catus	85.5	85.8	85.0	86.0		86.8	74.6	74.4	62.2	61.1	61.9
F	Mustela putorius	84.5	84.7	83.9	84.7	91.2		73.8	72.0	62.4	61.1	62.2
G	Loxodonta africana	84.7	85.0	84.2	83.4	85.2	83.4		72.3	62.4	60.4	60.4
H	Bos taurus	85.8	86.0	85.0	85.8	86.3	83.9	85.0		59.8	59.6	59.3
I	Rattus norvegicus	75.4	75.6	76.7	77.2	74.4	73.8	74.4	74.1		81.6	72.5
J	Mus musculus	76.9	77.2	77.5	78.2	75.6	74.1	74.1	74.4	89.6		72.8
K	Cricetulus griseus	75.1	75.1	76.2	74.9	75.1	74.1	73.1	72.8	81.9	84.5	

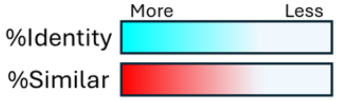

Supplementary Figure 1. SKA3 protein alignments, identity, and similarity of various vertebrate species performed by DNASTAR MegAlignPro Version: 18.1.1 (8) using MUSCLE. (A) Residue depictions colored by chemistry. (B) Percent identity and similarity indicated numerically and colorimetrically by blue and red, respectively.

bioRxiv preprint doi: https://doi.org/10.64898/2026.05.22.727258; this version posted May 24, 2026. The copyright holder for this preprint (which was not certified by peer review) is the author/funder, who has granted bioRxiv a license to display the preprint in perpetuity. It is made available under a CC-BY 4.0 International license.

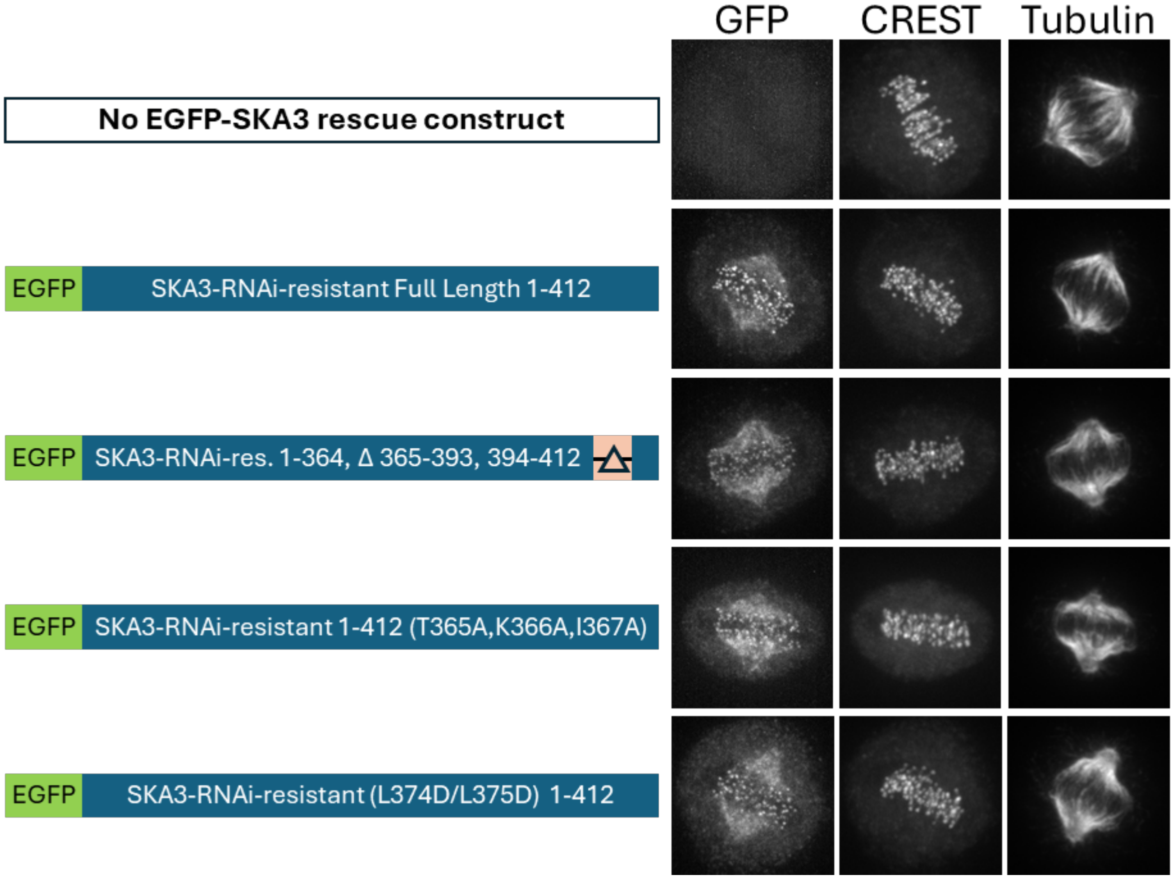

Supplementary Figure 2. Immunofluorescence panels showing localization of expressed RNAi-resistant EGFP-SKA3 constructs via anti-GFP staining, CREST, and Tubulin in HeLa cells.

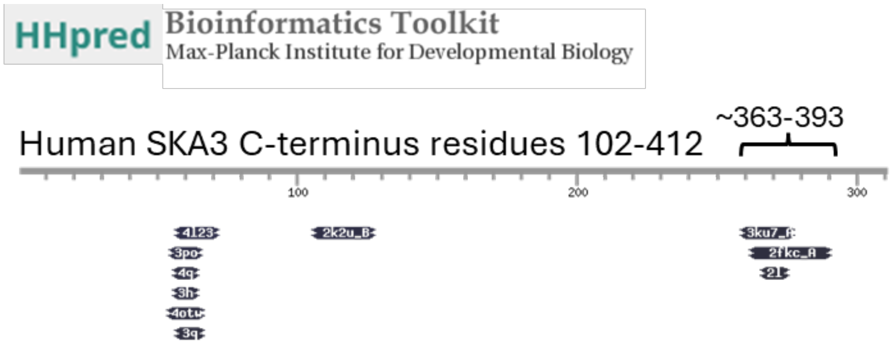

Supplementary Figure 3. HHpred predictions showing potential structural similarity within the C-terminal residues 102-412 of human SKA3 protein with that of known but likely unrelated proteins suggesting ordered, secondary protein structure may exist within the largely disordered SKA3 C-terminus. A region from roughly amino acids 363 through 393 of SKA3 is accentuated.

bioRxiv preprint doi: https://doi.org/10.64898/2026.05.22.727258; this version posted May 24, 2026. The copyright holder for this preprint (which was not certified by peer review) is the author/funder, who has granted bioRxiv a license to display the preprint in perpetuity. It is made available under a CC-BY 4.0 International license.

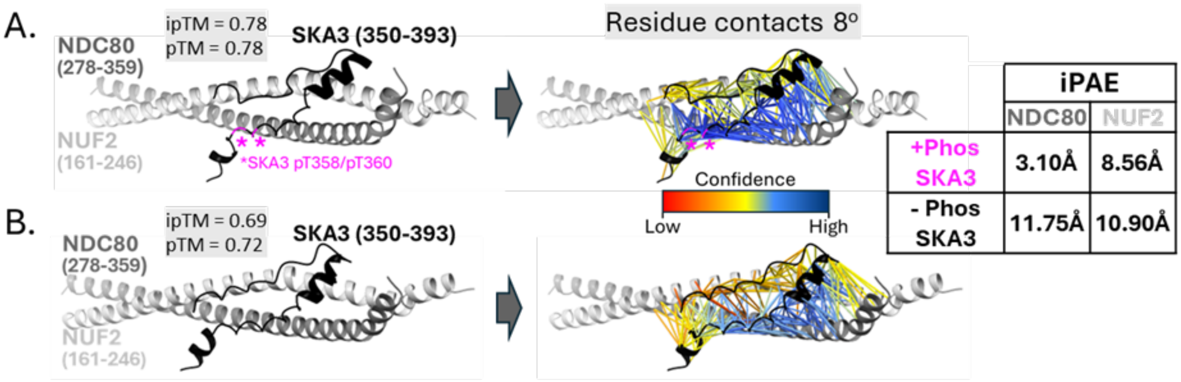

	iPAE	
	NDC80	NUF2
+Phos SKA3	3.10Å	8.56Å
- Phos SKA3	11.75Å	10.90Å

Supplementary Figure 4. AlphaFold3-predicted structural protein models of NDC80/NUF2 coiled-coil fragment and SKA3's C-terminal residues 350-393 with or without SKA3 phosphorylated residues. ipTM and pTM confidence metrics are shown. Residue contacts of PAE ≤8Å with colorimetric confidence scale and iPAE are reported. (A) Phosphorylated pT358 and pT360 SKA3 residues are magenta. (B) SKA3 modeled without phosphorylated residues.
